# Red Sea *Suberea mollis* Sponge Extract Protects against CCl_4_-Induced Acute Liver Injury in Rats via an Antioxidant Mechanism

**DOI:** 10.1155/2014/745606

**Published:** 2014-08-19

**Authors:** Aymn T. Abbas, Nagla A. El-Shitany, Lamiaa A. Shaala, Soad S. Ali, Esam I. Azhar, Umama A. Abdel-dayem, Diaa T. A. Youssef

**Affiliations:** ^1^Special Infectious Agents Unit, King Fahd Medical Research Centrer, King Abdulaziz University, Jeddah 21589, Saudi Arabia; ^2^Biotechnology Research Laboratories, Gastroenterology Surgery Center, Mansoura University, Mansoura 35516, Egypt; ^3^Department of Pharmacology and Toxicology, Faculty of Pharmacy, King Abdulaziz University, Jeddah 21589, Saudi Arabia; ^4^Department of Pharmacology and Toxicology, Faculty of Pharmacy, Tanta University, Tanta 31527, Egypt; ^5^Natural Products Unit, King Fahd Medical Research Center, King Abdulaziz University, Jeddah 21589, Saudi Arabia; ^6^Anatomy Department (Cytology and Histology), Faculty of Medicine, King Abdulaziz University, Jeddah 21589, Saudi Arabia; ^7^Medical Laboratory Technology Department, Faculty of Applied Medical Sciences, King Abdulaziz University, Jeddah 21589, Saudi Arabia; ^8^Animal Facility Unit, King Fahd Medical Research Center, King Abdulaziz University, Jeddah 21589, Saudi Arabia; ^9^Department of Natural Products, Faculty of Pharmacy, King Abdulaziz University, Jeddah 21589, Saudi Arabia

## Abstract

Recent studies have demonstrated that marine sponges and their active constituents exhibited several potential medical applications. This study aimed to evaluate the possible hepatoprotective role as well as the antioxidant effect of the Red Sea *Suberea mollis* sponge extract (SMSE) on carbon tetrachloride- (CCl_4_-) induced acute liver injury in rats. In vitro antioxidant activity of SMSE was evaluated by 2,2-diphenyl-1-picryl-hydrazyl-hydrate (DPPH) assay. Rats were orally administered three different concentrations (100, 200, and 400 mg/kg) of SMSE and silymarin (100 mg/kg) along with CCl_4_ (1 mL/kg, i.p., every 72 hr) for 14 days. Plasma aspartate aminotransferase (AST), alanine aminotransferase (ALT), alkaline phosphatase (ALP), and total bilirubin were measured. Hepatic malondialdehyde (MDA), reduced glutathione (GSH), nitric oxide (NO), superoxide dismutase (SOD), glutathione peroxidase (GPx), and catalase (CAT) were also measured. Liver specimens were histopathologically examined. SMSE showed strong scavenging activity against free radicals in DPPH assay. SMSE significantly reduced liver enzyme activities. Moreover, SMSE significantly reduced hepatic MDA formation. In addition, SMSE restored GSH, NO, SOD, GPx, and CAT. The histopathological results confirmed these findings. The results of this study suggested a potent protective effect of the SMSE against CCl_4_-induced hepatic injury. This may be due to its antioxidant and radical scavenging activity.

## 1. Introduction

Hepatotoxicity is a prevalent problem worldwide and represents 38% of all hepatic problems [1]. It is known that carbon tetrachloride (CCl_4_) causes acute liver toxicity in humans and experimental animals [2, 3]. Hepatotoxicity using CCl_4_ is a common model used to measure the efficiency of several antihepatotoxic drugs [4]. There are many previous in vivo and in vitro studies that documented the mechanism of CCl_4_-induced hepatocyte damage [5]. CCl_4_ is converted by cytochrome P450 (CYP) 2E1 to trichloromethyl (CCl_3_^•^) free radical and trichloromethylperoxy radical (CCl_3_OO^•^). Both radicals covalently bond to cellular macromolecules, producing lipid peroxidation, protein degeneration, DNA damage, and apoptosis [4–6].

Marine sponges are considered to be a gold mine because of their diversity of secondary vital biological compounds, which are not present in terrestrial organisms [7, 8]. Many worldwide diseases could be treated by drugs extracted from the sponges [9]. Marine sponges of the order Verongida, including genus* Suberea*, attracted the attention of chemists specializing in marine-derived natural products. They possess an unusual chemical structure due to large amounts of sterols and a lack of terpenes and typical brominated compounds associated with tyrosine [10]. Members of the genus* Suberea* display diverse bioactivities, including antibacterial [11], antiviral [12], enzyme inhibition [13], and cytotoxic activity [14]. Prenylated toluquinone, hydroquinones, and naphthoquinones are examples of marine-derived natural products with reported antioxidant activities [15–18].

The present study aims to evaluate the possible hepatoprotective effect of the Red Sea* Suberea mollis* sponge extract (SMSE) against CCl_4_-induced hepatotoxicity as compared to silymarin, the most commonly known hepatoprotective agent. In addition, the mechanism of the suggested effect was studied regarding potential antioxidant properties of the organic extract of the sponge.

## 2. Materials and Methods

### 2.1. Sponge Collection

**Figure 1:**
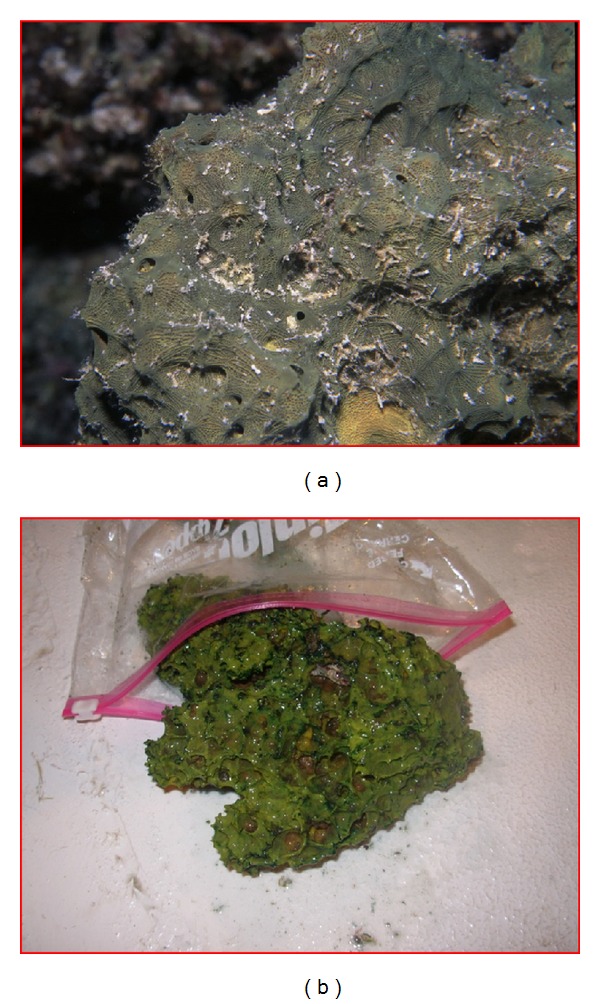
Underwater (a) and in situ (b) photographs of the* Suberea mollis* sponge.

The sponge was collected from the Red Sea in 2011, between depths of 15 and 25 meters. The sponge was cylindrical in shape and had a low and a sharp conulose surface. The sharpness of the conules was due to the projection of strong fibers about 8–10 mm apart (Figure 1). The diameter of the oscules was about 1.0 cm, and they were located at the summit of the fragment. The interior of the sponge was cavernous. The fresh sponge had a green color with a yellowish interior, while the preserved sponge had a black color. The fresh sponge was frozen immediately after collection.

### 2.2. Identification of the Sponge

The sponge was kindly identified by Professor Rob van Soest at Naturalis Biodiversity Center, Department of Marine Zoology, RA Leiden, The Netherlands. The voucher specimen, measuring 3.5 cm, is incorporated in the collections of the Zoological Museum of the University of Amsterdam under registration number 16621. Another voucher specimen was deposited in the Red Sea Invertebrates Collection of the Department of Pharmacognosy, Suez Canal University, under the code number DY-8.

### 2.3. Extraction of the Sponge

The extraction was done using methanol (3 × 3000 mL) at room temperature after crushing the frozen sponge. The combined crude extracts evaporated under the reduced pressure.

### 2.4. Determination of In Vitro Antioxidant Activity (DPPH Test)

Different concentrations of the SMSE extract were dissolved in ethanol. 2 mL of each concentration was pipetted into a series of 5 mL volumetric flasks. 3 mL of DPPH (2,2-diphenyl-1-picryl-hydrazyl-hydrate) solution was added to each flask and mixed with extract solution. Methanol was used as a blank. The absorbance was measured at 517 nm after 10-minute incubation [19] using a Thermo Scientific GENESYS 10S UV-visible double beam spectrophotometer (USA). *α*-Tocopherol (vitamin E) was used as the positive control [20]. Higher free radical scavenging activity was indicated by lower absorbance of the reaction mixture. The following equation [20] shows the radical scavenging activity of the sample, which is expressed as the inhibition percentage: % inhibition = [(Ac −A sample)/Ac] × 100, where Ac is the absorbance of the control (DPPH in absence of extract) and A sample is the absorbance in the presence of the extract. All of the tests were performed in triplicate.

### 2.5. Animals

Male albino Sprague Dawley rats (200–220 g) were obtained from the Animal Resources Division of King Fahd Medical Research Center. The rats were housed at 22 ± 3°C and relative humidity of 44%–55% with a 12 h dark/light cycle and were provided with standard laboratory feed and water ad libitum. The use of experimental animals in this study was conducted under the guidance of the basic standards in the care and use of laboratory animals, which has been prepared and published by the National Institutes of Health. The study protocol has been approved by the Research Ethics Committee at King Abdulaziz University (Approval number 29-14).

### 2.6. Estimation of SMSE Dose

The extract (300 mg/kg) was orally administered to three rats, which were fasted overnight. The animals were observed daily for 14 days for mortality [21]. The procedure was repeated for higher doses up to 2000 mg/kg bw. 1/20, 1/10, and 1/5 of highly tolerated doses (2000 mg/kg) were selected (100, 200, and 400 mg/kg, resp.) for assessment of hepatoprotective activity [22].

### 2.7. Experimental Design

Three different doses of the SMSE (100, 200, and 400 mg/kg) were tested for their hepatoprotective effect against CCl_4_-induced hepatotoxicity. A total of 30 rats were divided into six groups (*n* = 5). (1) Control: rats in this group were orally administered 1 mL/kg of dimethyl sulfoxide (DMSO) daily for 14 days. (2) CCl_4_: this group served as the model of hepatotoxicity. The rats in this group were intraperitoneally (i.p.) administered CCl_4_ in olive oil (1 mL/kg, 1 : 1 v/v) every 72 hours for 14 days [23]. (3) CCl_4_ + silymarin (100 mg/kg): this group served as a positive control. Rats in this group were administered silymarin (Sigma Chemicals Company, USA) (100 mg/kg, orally through a feeding tube) in DMSO daily for 14 days [24]. CCl_4_ in olive oil was also given to this group every 72 hours for 14 days. (4) CCl_4_ + SMSE (100 mg/kg): SMSE (100 mg/kg) dissolved in DMSO was given orally daily for 14 days, and CCl_4_ in olive oil (1 mL/kg, i.p. 1 : 1 v/v) was given every 72 hours for 14 days. (5) CCl_4_ + SMSE (200 mg/kg): SMSE (200 mg/kg) dissolved in DMSO was given orally daily for 14 days, and CCl_4_ in olive oil (1 mL/kg, i.p. 1 : 1 v/v) was given every 72 hours for 14 days. (6) CCl_4_ + SMSE (400 mg/kg): SMSE (400 mg/kg) dissolved in DMSO was given orally daily for 14 days, and CCl_4_ in olive oil (1 mL/kg, i.p. 1 : 1 v/v) was given every 72 hours for 14 days. Two days after the last dose, blood from all of the rats was collected via retroorbital sinus plexus under mild ether anesthesia [24]. Rats were sacrificed by cervical dislocation. Blood was allowed to clot at room temperature and the serum was separated by centrifuging at 4000 rpm for 15 min and was kept at −20°C for further biochemical analysis. The liver was dissected and used for histopathological (formalin fixed) and biochemical (frozen −80°C) studies.

### 2.8. Determination of Alanine Amino Transaminase (ALT), Aspartate Amino Transaminase (AST), Alkaline Phosphatase (ALP), and Total Bilirubin

Various liver marker enzymes, such as ALT, AST, ALP, and total bilirubin, were measured in plasma using an automated analyzer (Flexor EL200, France).

### 2.9. Sample Preparation for Biochemical Analysis

The liver samples were homogenized in 2% Triton X-100 containing 0.32 M sucrose solution for SOD determination. Other liver portions were homogenized in 50 Mm potassium phosphate pH 7.5 and 1 Mm EDTA for MDA, GSH, NO, GPx, and CAT measurements. Homogenized tissues were subjected to a sonication procedure twice with 30 s intervals at 4°C. After the sonication process, homogenized tissues were centrifuged at 4000 rpm/min for 10 min at 4°C [25].

### 2.10. Determination of Lipid Peroxide (Measured as MDA)

MDA was determined in the liver homogenates using kits provided by Biodiagnostic, Egypt. MDA was determined according to the method of Uchiyama and Mihara [26]. The adducts were formed following the reaction of thiobarbituric acid with tissue homogenate in a boiling water bath and were extracted with n-butanol. Tissue MDA content was measured by the difference in optical density developed at two distinct wavelengths, 535 nm and 525 nm. Tissue MDA content was expressed as nmol/g tissue.

### 2.11. Determination of Reduced Glutathione (GSH)

GSH was determined in the liver homogenates using kits provided by Biodiagnostic, Egypt. GSH was determined according to the method described earlier by Ellman [27]. The principle of this procedure is based on the formation of 2-nitro-5-mercaptobenzoic acid from reduction of bis(3-carboxy-4-nitrophenyl) disulfide reagent by the SH group, which has a deep yellow color that was measured spectrophotometrically at 412 nm [28]. Tissue GSH content was expressed as nmol/g tissue.

### 2.12. Determination of Nitric Oxide (NO)

NO was determined in the liver homogenates using kits provided by Biodiagnostic, Egypt. Initially, nitrate was converted into nitrite by the enzyme nitrate reductase, followed by quantitation of nitrite using Griess reagent at the absorbance of 550 nm, as previously described [29]. NO was assayed by measuring total nitrate plus nitrite (NO_3_^−^ + NO_2_^−^), the stable end products of NO metabolism. Results were expressed as *μ*mol/g tissue.

### 2.13. Determination of Glutathione Peroxidase (GPx)

GPx activity was determined in the liver homogenates using kits provided by Biodiagnostic, Egypt. GPx activity was determined in a coupled assay with glutathione reductase by measuring the rate of NADPH oxidation at 340 nm using H_2_O_2_ as the substrate [30]. GPx activity was expressed in mU/g tissue.

### 2.14. Determination of Superoxide Dismutase (SOD)

The activity of SOD was determined in the liver homogenates using kits provided by Biodiagnostic, Egypt. SOD was determined according to the method described earlier by Nishikimi et al. [31]. This assay relies on the ability of the enzyme to inhibit the phenazine methosulphate-mediated reduction of nitroblue tetrazolium dye. SOD activity was expressed in U/g tissue.

### 2.15. Determination of Catalase (CAT)

The activity of CAT was determined in the liver homogenates using kits provided by Biodiagnostic, Egypt. This enzyme was measured according to Aebi [32]. H_2_O_2_ reacts with a known quantity of CAT. After adding catalase inhibitor, the reaction was stopped after exactly one minute. The remaining H_2_O_2_ reacts with 3,5-dichloro-2-hydroxybenzene sulfonic acid (DHBS) and 4-aminophenazone (AAP) to form a chromophore with color intensity inversely proportional to the amount of CAT in the original sample. The absorbance of samples was read at 510 nm against a standard blank. CAT activity was expressed in U/g tissue.

### 2.16. Histopathological Examination

For a histopathological study of the liver, the animals were dissected via abdominal incision, and the livers of all groups were extracted. Slices of liver 2 × 2 cm were excised and fixed in 10% neutral buffered formalin for further processing using the paraffin technique. Five-micron paraffin sections were stained with hematoxylin and eosin (H&E) and examined by a light microscope connected to a digital camera. Photographs of the liver at different magnifications were screened for features of hepatotoxicity and the hypothesized protective efficacy.

### 2.17. Statistical Analysis

All data were presented as mean ± SD. The results were statistically analyzed by an ANOVA test utilizing SPSS10.0 statistical software. Statistical differences of *P* ≤ 0.05 were considered significant.

## 3. Results

### 3.1. Effect of SMSE on In Vitro Antioxidant Activity (DPPH Test)

**Figure 2:**
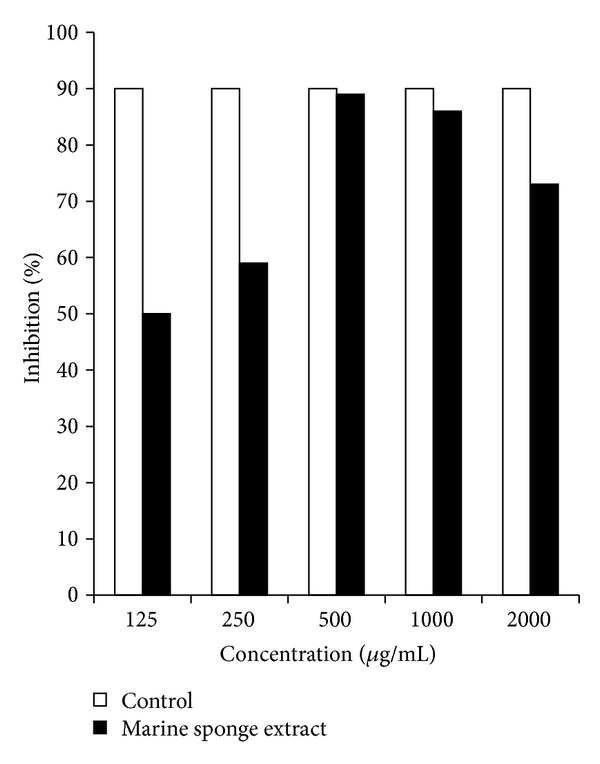
Total antioxidant capacity of different concentrations of* Suberea mollis* sponge extract (SMSE). Expressed as percent inhibition toward DPPH-induced oxidative stress in vitro.

The scavenging activity of SMSE (125, 250, 500, 1000, and 2000 *μ*g/mL) against DPPH free radicals was demonstrated in Figure 2. SMSE had a strong DPPH radical inhibition and their percentage of inhibition (89%) nearly reached the control percentage inhibition (90%) at 500 and 1000 *μ*g/mL.

### 3.2. Effect of SMSE and Silymarin on Liver Functions Measured as ALT, AST, ALP, and Bilirubin

The results of the liver function tests are shown in Table 1. Treatment of rats with CCl_4_ caused a significant increase in ALT by 266%, AST by 57.47%, ALP by 152.91%, and total bilirubin by 126.6% compared to the control group (*P* = 0.001). Treatment of CCl_4_-injected rats with silymarin significantly decreased all measured serum biochemical activities (ALT 60.75%, AST 29.87%, ALP 52.41%, and total bilirubin 51.47%) compared to the CCl_4_ group (*P* = 0.001). Treatment of CCl_4_-injected rats with the SMSE (100 mg/kg) significantly decreased the percentage of liver marker enzymes and total bilirubin: ALT 47.32% (*P* = 0.001), AST 12.67%, ALP 29.78% (*P* = 0.001), and total bilirubin 42.27% (*P* = 0.001) compared to the CCl_4_ group. On the other hand, the percentage protection was increased at the dose of 200 and 400 mg/kg: ALT 56.11% (*P* = 0.001), 71.56% (*P* = 0.001); AST 26% (*P* = 0.01), 31.18% (*P* = 0.001); ALP 41.77% (*P* = 0.001), 45.13% (*P* = 0.001); and total bilirubin 53.3% (*P* = 0.001), 54% (*P* = 0.001), respectively.

### 3.3. Effect of SMSE and Silymarin on CCl_4_-Induced Changes in Liver MDA, GSH, and NO

**Figure 3:**
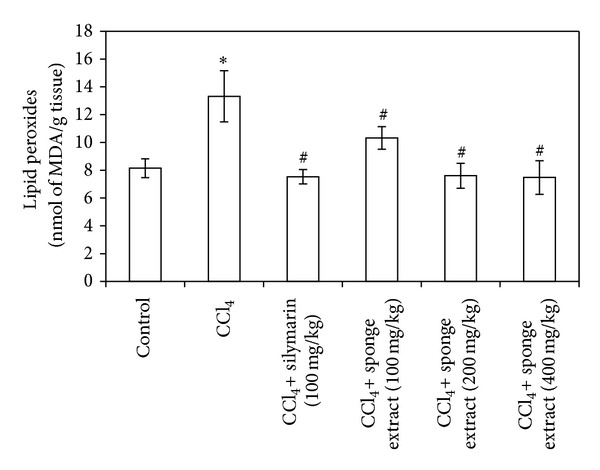
Effect of* Suberea mollis* sponge extract (SMSE) and silymarin on liver lipid peroxides in CCl_4_-induced hepatotoxicity in rats. The effect of SMSE (100, 200, and 400 mg/kg) and silymarin (100 mg/kg) on liver lipid peroxides (measured as MDA) concentration measured in CCl_4_-induced hepatotoxicity in rats. Each point represents the mean ± SD of five rats. ∗Significant difference compared with the control group (*P* ≤ 0.05). ^#^Significant difference compared with the CCl_4_ group (*P* ≤ 0.05).

**Figure 4:**
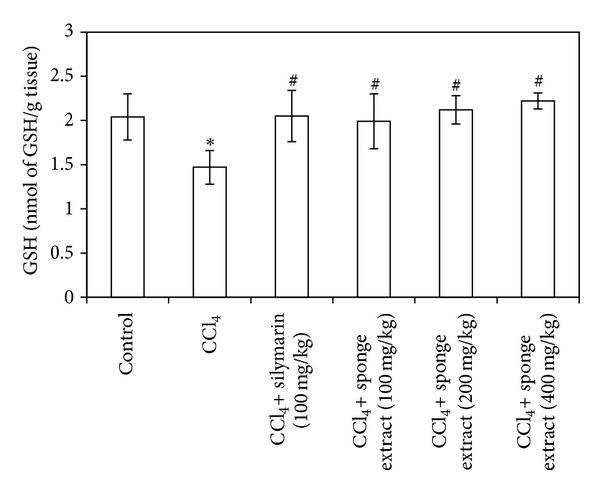
Effect of* Suberea mollis* sponge extract (SMSE) and silymarin on liver GSH in CCl_4_-induced hepatotoxicity in rats. The effect of SMSE (100, 200, and 400 mg/kg) and silymarin (100 mg/kg) on liver GSH contents measured in CCl_4_-induced hepatotoxicity in rats. Each point represents the mean ± SD of five rats. ∗Significant difference compared with the control group (*P* ≤ 0.05). ^#^Significant difference compared with the CCl_4_ group (*P* ≤ 0.05).

**Figure 5:**
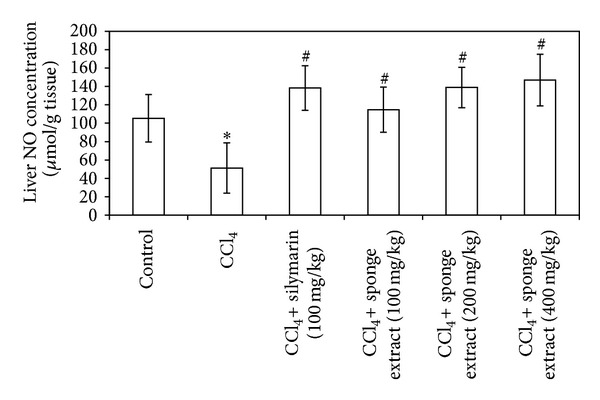
Effect of* Suberea mollis* sponge extract (SMSE) and silymarin on liver NO in CCl_4_-induced hepatotoxicity in rats. The effect of SMSE (100, 200, and 400 mg/kg) and silymarin (100 mg/kg) on liver NO concentration measured in CCl_4_-induced hepatotoxicity in rats. Each point represents the mean ± SD of five rats. ∗Significant difference compared with the control group (*P* ≤ 0.05). ^#^Significant difference compared with the CCl_4_ group (*P* ≤ 0.05).

The treatment of rats with CCl_4_ caused a significant increase (63%) in liver MDA contents compared to the control group (*P* = 0.002) (Figure 3). On the other hand, the treatment of rats with CCl_4_ caused a significant decrease (28% and 51%) in liver GSH and NO contents, respectively, compared to the control group (*P* = 0.012 and 0.019) (Figures 4 and 5). Treatment of CCl_4_-injected rats with silymarin significantly decreased (43%) liver MDA contents and increased (28% and 170%) liver GSH and NO contents, respectively, compared to the CCl_4_ group (*P* = 0.001, 0.016, and 0.004) (Figures 3, 4, and 5). Treatment of CCl_4_-injected rats with the SMSE (100, 200, and 400 mg/kg) significantly decreased (22%, 42%, and 43%) liver MDA contents, respectively, compared to the CCl_4_ group (*P* = 0.024, 0.001, and 0.002) (Figure 3). In addition, treatment of CCl_4_-injected rats with the SMSE (100, 200, and 400 mg/kg) significantly increased liver contents of both GSH (35%, 44%, and 51%) (*P* = 0.029, 0.002, and 0.000) and NO (124%, 171%, and 187%) (*P* = 0.005, 0.001, and 0.001), respectively, compared to the CCl_4_ group (Figures 4 and 5).

### 3.4. Effect of SMSE and Silymarin on CCl_4_-Induced Changes in Liver GPx, SOD, and CAT Activities

The results of enzymatic antioxidant analyses are shown in Table 2. Briefly, the activities of GPx, SOD, and CAT were significantly decreased (78%, 39%, and 41%) in the CCl_4_ treated group compared to the control group (*P* = 0.002, 0.05, and 0.002). On the other hand, treatment of CCl_4_-injected rats with either silymarin or SMSE (100, 200, and 400 mg/kg) significantly increased GPx (3-, 6-, 6-, and 8-fold) (*P* = 0.011, 0.004, 0.008, and 0.000), respectively, compared to the CCl_4_ group. Treatment of CCl_4_-injected rats with either silymarin or SMSE (400 mg/kg) significantly increased SOD (59% and 190%) (*P* = 0.02 and 0.001), respectively, compared to the CCl_4_ group. In addition, treatment of CCl_4_-injected rats with either silymarin or SMSE (200 and 400 mg/kg) significantly increased CAT (73%, 59%, and 65%) (*P* = 0.002, 0.007, and 0.006), respectively, compared to the CCl_4_ group.

### 3.5. Histopathological Microscopic Study

**Figure 6:**
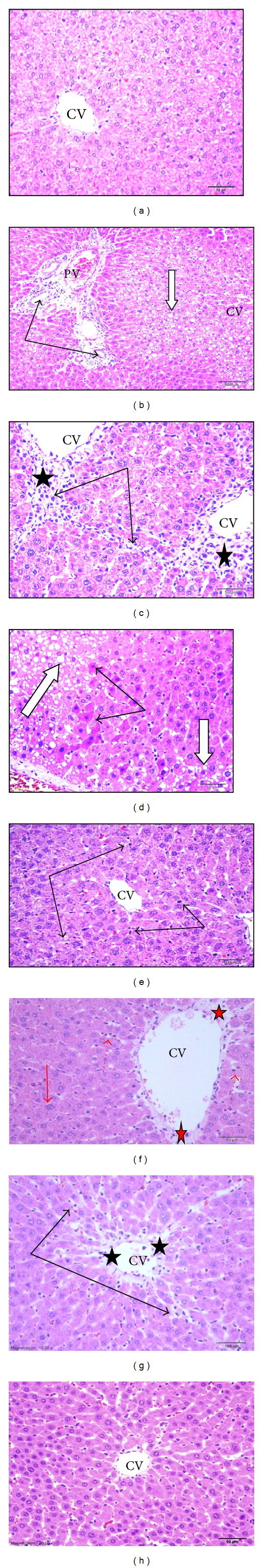
Histopathological study of liver tissue in control, CCl_4_, silymarin, and* Suberea mollis* sponge extract (SMSE) groups of rats. (a) Control group showed normal liver architecture. (b, c, and d) CCl_4_ group: (b) showed portal areas with dilated congested vein (PV) and proliferating bile ducts (arrows) and inflammatory cells. Notice fatty infiltration of hepatocytes (white arrow). (c) revealed cell necrosis (stars) near central veins (CV) with fibrous bridging (thin arrows). (d) showed increased dark degenerated apoptotic hepatocytes (black arrows) and fatty infiltration (white arrow). (e) CCl_4_ + silymarin (100 mg/kg) showed normal hepatocytes around the central vein with an absence of fatty infiltration. Few cells showed karyomegaly and dark degenerated nuclei (black arrow). (f) CCl_4_ + SMSE (100 mg/kg) showed normal hepatocytes around the central vein (CV) with few cells showing karyomegaly (red arrows). Blood sinusoids showed lymphocyte (dotted arrows) infiltration. Mild perivenular fibrosis (star) could be seen. (g) CCl_4_ + SMSE (200 mg/kg) showed normal hepatocytes (black arrows) with no signs of fatty changes around the central vein (CV) and mild perivenular fibrosis (star). (h) CCl_4_ + SMSE (400 mg/kg): region near central vein (CV) showed normal hepatocytes with an absence of any signs of fatty infiltration. Absence of perivenular fibrosis (H&E stain).

The histological architecture of the control rat livers was similar to normal rat livers (Figure 6(a)). CCl_4_ administration caused central perivenular cell necrosis and fibrous bridging. Bile duct proliferation, portal vein congestion, and inflammatory cell infiltrate were also observed. Karyomegaly and fatty infiltration of hepatocytes were observed in central regions of the hepatic lobules. Some samples showed increased degeneration of apoptotic cells (Figures 6(b), 6(c), and 6(d)). Silymarin provided protection against fatty infiltration, nuclear changes, and bile duct proliferation (Figure 6(e)). Administration of 100 mg/kg SMSE resulted in potential protection against fatty infiltration. Lymphocytes were frequent within hepatic sinusoids (Figure 6(f)). On the other hand, histopathological findings showed that protection of rat liver against CCl_4_ hepatotoxicity was more profound in rats that received 200 mg/kg of SMSE when compared to the previous low dose group. There was an absence of fatty infiltration, and portal changes and apoptotic hepatocytes were less frequent (Figure 6(g)). Liver sections of rats treated with SMSE at a dose of 400 mg/kg showed healthy hepatocytes with active vesicular nuclei. Signs of fatty changes, fibrosis, or portal tract changes were not observed (Figure 6(h)).

## 4. Discussion

The data presented in this study demonstrate that the crude SMSE protected against CCl_4_-induced liver injury. This was reflected by the significant decrease in serum levels of ALT, AST, ALP, and total bilirubin and histopathologically by the protection against CCl_4_-induced liver degeneration. Similarly, Abdel-Monem et al. [33] recently found that* Trichurus spiralis* extract isolated from* Hippospongia communis* sponges possesses a hepatoprotective activity against heavy-metal-mixture-induced liver damage. It is clear that the hepatotoxicity of CCl_4_ increased with both the depletion of GSH and the increase of free radicals and lipid oxidation [34, 35]. In the present study, the treatment of rats with CCl_4_ resulted in the depletion of hepatic GSH and NO and a decrease in the activity of GPx, SOD, and CAT. It also increased the liver lipid peroxidation product, MDA. This observation was in agreement with the recent findings of Simeonova et al. [36]. On the other hand, pretreatment with SMSE resulted in a significant decrease of MDA quantity and increase in GSH content, as well as a significant increase in the activity of GPx, SOD, and CAT. GSH serves as the main cytosolic antioxidants; it has a central role in sulfhydryl homeostasis [37, 38]. Recently, Lind et al. [39] found that barettin isolated from the marine sponge* Geodia barretti* possesses antioxidant properties in ferric reducing antioxidant power (FRAP) assays and lipid peroxidation cell assays. In addition, Dupont et al. [40] reported that the crude extract of the marine sponge* Phorbastenacior* exhibited effects against DPPH-induced oxidative stress.* Suberea mollis* in the order Verongida in the family Aplysinellidae afforded two new brominated arginine-derived alkaloids: subereamines A and B; a new brominated phenolic compound has exhibited strong antioxidant potential compared to vitamin E and ascorbic acid; moreover,* Suberea mollis* contain subereaphenol D and the known compounds dichloroverongiaquinol, aerothionin, and purealdin L [41, 42]. Subereaphenol was reported to be a free radical scavenger in the DPPH assay [41, 42]. Previous findings are consistent with the scavenging activity of SMSE against DPPH-induced oxidative stress in this study, which reaches 89%, near to the standard antioxidant control (90%). The result suggested that SMSE at a concentration of 500 *μ*g/mL was found to have the highest inhibitory activity against DPPH. However, the decrease in radical scavenging activity was observed with increasing SMSE concentration (1000 and 2000 *μ*g/mL) indicating a possible prooxidative effect of SMSE at high concentration. This result was in accordance with Kiokias and Gordon [43] and Chen and Yen [44], who found the autoxidation of carotenoids and guava leaf extracts, respectively, at a high concentration in vitro.

Nitric oxide (NO) is an important biological mediator and has been shown to be involved in diverse physiological and pathological processes [45]. It has various functional messengers in numerous vertebrates. The liver is an organ that is clearly influenced by NO [46]. Using different models of liver damage, the role of NO in the liver remains controversial and it may be associated with both beneficial and detrimental consequences [47]. NO was found to have a protective role in mild oxidative hepatotoxicity models, although this was not the case in severe hepatotoxicity models. In the hepatocytes, there is a balance between the generation rate of NO and its degradation. The disturbance in this balance led to harmful free radical formation, which may be responsible for liver toxicity [45]. NO is a highly reactive oxidant synthesized from L-arginine by inducible nitric oxide synthase (NOS II) and produced from parenchymal and nonparenchymal liver cells [48, 49]. In this study, the treatment of rats with CCl_4_ led to a significant unexpected decrease of liver total nitrates contents compared to the rats in the control group. In addition, the histopathological results showed enhanced severe liver damage, suggesting that the decreased NO release in the liver is attributable to CCl_4_-induced hepatotoxicity. Similarly, Laskin et al. [50] found that CCl_4_-induced hepatotoxicity was increased in mice lacking the gene for NO synthesis. On the other hand, pretreatment of rats with SMSE and silymarin resulted in a significant increase in liver NO content. Previous data suggested that the hepatoprotective effects of nitric oxide in this model may be due in part to the inhibition of TNF-α [51]. The attenuation of metal/peroxide oxidative chemistry, as well as lipid peroxidation, appears to be the major chemical mechanisms by which NO may limit oxidative injury [52].

## 5. Conclusion

This study suggested a protective effect of the extract of SMSE against CCl_4_-induced hepatotoxicity. The mechanisms for the hepatoprotective effect center on the increased GSH synthesis, increased GPx, CAT, and SOD activities, and decreased MDA generation. In addition, a potential mechanism for the hepatoprotective role of the crude SMSE against CCl_4_-induced hepatotoxicity may center on its role in NO homeostasis.

## Figures and Tables

**Table 1:** Effects of the *Suberea mollis* sponge extract (SMSE) and silymarin on ALT, AST, ALK, and total bilirubin levels measured in CCl_4_-induced hepatotoxicity in rats.

Treatment regimen	ALT	AST	ALP	Total bilirubin
Control	44.2 ± 5.89	145.8 ± 13.08	123.6 ± 24.35	1.2 ± 0.45
CCl_4_	161.8 ± 13.71^a^	229.6 ± 19.48^a^	312.6 ± 49.06^a^	2.72 ± 0.19^a^
CCl_4_ + silymarin (100 mg/kg)	63.5 ± 18.59^b^	161 ± 29.8^b^	148.75 ± 37.6^b^	1.32 ± 0.12^b^
CCl_4_ + SMSE (100 mg/kg)	85.25 ± 15.2^b^	200.5 ± 34.8	219.5 ± 41.34^b^	1.57 ± 0.3^b^
CCl_4_ + SMSE (200 mg/kg)	71 ± 13.31^b^	169.7 ± 37.52^b^	182 ± 16.58^b^	1.27 ± 0.2^b^
CCl_4_ + SMSE (400 mg/kg)	46 ± 16^b^	158 ± 13.56^b^	171.5 ± 14.7^b^	1.25 ± 0.5^b^

Data are mean ± SD of five animals.

^a^Significantly different from control group (*P* ≤ 0.05).

^b^Significantly different from the CCl_4_ group (*P* ≤ 0.05).

**Table 2:** Effect of *Suberea mollis* sponge extract (SMSE) and silymarin on liver GPx, SOD, and CAT measured in CCl_4_-induced hepatotoxicity in rats.

Treatment regimen	GPx (mU/g tissue)	SOD (U/g tissue)	CAT (U/g tissue)
Control	24.8 ± 6.9	18.3 ± 4.8	8.2 ± 0.2
CCl_4_	5.3 ± 9.2^a^	11.13 ± 3.6^a^	4.8 ± 1.3^a^
CCl_4_ + silymarin (100 mg/kg)	15.5 ± 4.7^b^	17.8 ± 2.3^b^	8.3 ± 0.3^b^
CCl_4_ + SMSE (100 mg/kg)	29.1 ± 10.1^b^	12.7 ± 2.8	4.5 ± 1.3
CCl_4_ + SMSE (200 mg/kg)	32.5 ± 13.6^b^	16.8 ± 3.6	7.65 ± 0.5^b^
CCl_4_ + SMSE (400 mg/kg)	42.3 ± 9.9^b^	32.7 ± 6.5^b^	7.9 ± 0.7^b^

Data are mean ± SD of five animals.

^a^Significantly different from control group (*P* ≤ 0.05).

^b^Significantly different from the CCl_4_ group (*P* ≤ 0.05).
